# Decipher the Glioblastoma Microenvironment: The First Milestone for New Groundbreaking Therapeutic Strategies

**DOI:** 10.3390/genes12030445

**Published:** 2021-03-20

**Authors:** Giuseppe Nicolò Fanelli, Dario Grassini, Valerio Ortenzi, Francesco Pasqualetti, Nicola Montemurro, Paolo Perrini, Antonio Giuseppe Naccarato, Cristian Scatena

**Affiliations:** 1 Division of Pathology, Department of Translational Research and New Technologies in Medicine and Surgery, University of Pisa, 56126 Pisa, Italy; gnfanelli@gmail.com (G.N.F.); dario.grassini23@gmail.com (D.G.); giuseppe.naccarato@unipi.it (A.G.N.); 2 Anatomia Patologica 1, Department of Laboratory Medicine, Pisa University Hospital, 56126 Pisa, Italy; valerio.ortenzi@ao-pisa.toscana.it; 3 Division of Radiation Oncology, Department of Medical and Oncological Area, Pisa University Hospital, 56126 Pisa, Italy; francep24@hotmail.com; 4 Division of Neurosurgery, Department of Translational Research and New Technologies in Medicine and Surgery, University of Pisa, 56126 Pisa, Italy; nicola.montemurro@unipi.it (N.M.); paolo.perrini@unipi.it (P.P.)

**Keywords:** glioblastoma, tumour microenvironment, immunotherapy, cancer stem-cells

## Abstract

Glioblastoma (GBM) is the most common primary malignant brain tumour in adults. Despite the combination of novel therapeutical approaches, it remains a deadly malignancy with an abysmal prognosis. GBM is a polymorphic tumour from both molecular and histological points of view. It consists of different malignant cells and various stromal cells, contributing to tumour initiation, progression, and treatment response. GBM’s microenvironment is multifaceted and is made up of soluble factors, extracellular matrix components, tissue-resident cell types (e.g., neurons, astrocytes, endothelial cells, pericytes, and fibroblasts) together with resident (e.g., microglia) or recruited (e.g., bone marrow-derived macrophages) immune cells. These latter constitute the so-called immune microenvironment, accounting for a substantial GBM’s tumour volume. Despite the abundance of immune cells, an intense state of tumour immunosuppression is promoted and developed; this represents the significant challenge for cancer cells’ immune-mediated destruction. Though literature data suggest that distinct GBM’s subtypes harbour differences in their microenvironment, its role in treatment response remains obscure. However, an in-depth investigation of GBM’s microenvironment may lead to novel therapeutic opportunities to improve patients’ outcomes. This review will elucidate the GBM’s microenvironment composition, highlighting the current state of the art in immunotherapy approaches. We will focus on novel strategies of active and passive immunotherapies, including vaccination, gene therapy, checkpoint blockade, and adoptive T-cell therapies.

## 1. Introduction

Brain tumours are characterized by high mortality due to their localization and invasive growth [1]. Glioblastoma (GBM) accounts for about 70% of gliomas and 15% of primary brain tumours, representing the most frequent primary malignant brain tumour in adults [2]. Recent attention to stem-like glioma cells has identified neural stem cells (NSCs), a subfraction of cells limited to germinal areas, as precursor cells in gliomas [3]. GBM consists of a heterogeneous population made of malignant cells and different stromal cells, which altogether contribute to tumour formation or progression and response to treatment [4]. The current standard of care of GBM contemplates surgical resection, radiotherapy, and chemotherapy with temozolomide (TMZ) [5]. Over the past few decades, improvements in outcome have been modest; however, new approaches (including gene therapy or targeted therapies—such as monoclonal antibodies or tyrosine kinase inhibitors together with immunotherapeutic strategies) may display more significant potential. In addition to efforts that have led to an improved understanding of the genetic basis of gliomas, a considerable body of work has indicated an increasing recognition of the interaction between glioma and immunity [6]. The significant survival advantage of immunotherapy in numerous extracranial tumours encouraged experimental studies in glioblastoma patients [7]. Data suggest that glioma cells develop mechanisms to evade the immune system [6]; in turn, the tumour microenvironment promotes a state of immunosuppression, offering new therapeutic chances that may improve the outcome of patients [4]. This review aims to elucidate the composition of GBM’s microenvironment and to highlight the current state of the art of novel immunotherapeutic approaches, with a particular focus on active and passive immunotherapies strategies, including vaccination, gene therapy, checkpoint blockade and adoptive T-cell therapies.

## 2. Epidemiology

Approximately 80% of all brain malignant tumours are gliomas, of which around 70% are GBM [8].

GBM occurs mainly in men with a median age of 64 years (1.3–1.6:1 compared to women), particularly in whites of European descent (2:1 compared to African Americans). The annual incidence ranges between 3 and 5 newly diagnosed cases per 100,000 inhabitants [9].

Notwithstanding the variety of current therapies, GBM remains a deadly disease with a tremendously poor prognosis: patients survive approximately 15 months after diagnosis, and only 5.5% of these are alive five years after diagnosis [10]. Clinical and biological parameters such as tumour size and location, age at presentation, Karnofsky performance score (KPS) at presentation, histologic findings and molecular genetic factors or therapeutic approaches undertaken affect survival in GBM patients [11].

## 3. Histological and Molecular Classification

According to morphological similarities with neuroglial cells in the normal brain, gliomas have been traditionally classified into astrocytomas, oligodendrogliomas, and ependymomas [9]. The World Health Organization (WHO) classifies astrocytomas based on histologic features into four grades of prognosis, of which anaplastic astrocytoma (grade III) and GBM (grade IV) are considered malignant gliomas [12]. GBM exhibits a pleomorphic cell population of small, poorly differentiated and large multinucleated tumour cells with brisk mitotic activity in the context of multifocal necrosis (with pseudopalisading nuclei) and glomeruloid microvascular proliferation [10]. Although GBM is one of the most heterogeneous neoplasms in humans, only three morphologic rare variants are recognized in the 2016 WHO Classification of Tumors of the Central Nervous System (CNS): gliosarcoma, giant cell glioblastoma, and epithelioid glioblastoma [13]. Malignant gliomas typically contain both neoplastic cells and stroma, contributing to the tumour’s histologic heterogeneity and variable outcome. Based on gene expression profiles, GBM can be further classified into four subtypes, such as classical, proneural, neural, and mesenchymal, conferring differences in terms of aggressiveness, potential to progress, and thus diverse prognosis to the tumour [14]. Histopathological classification is currently assisted by molecular genetic studies that provide diagnostic, prognostic, and predictive values [14]. In detail, three markers have undergone extensive studies in recent years: (i) 1p/19q chromosomal codeletion; (ii) O6-methylguanine methyltransferase (MGMT) promoter methylation; and (iii) mutations of isocitrate dehydrogenase (IDH) 1 and 2 [15]. According to the expression of these markers, gliomas of adulthood are classified into three main groups: (i) IDH-mutant, 1p19q co-deleted tumours, with a predominantly oligodendroglial morphology and favourable outcome; (ii) IDH-mutant, non-1p19q co-deleted tumours, with an astrocytic morphology and intermediate outcome; and (iii) IDH wild-type tumours, which are mostly glioblastomas, with an unfavourable prognosis [16]. In the 2016 WHO Classification of Tumors of the CNS, GBMs have been classified as: (i) glioblastoma IDH-wildtype (about 90% of cases), which corresponds to the clinically defined primary or de novo glioblastoma and occurs in patients over 55 years of age; (ii) glioblastoma IDH-mutant (about 10% of cases), which corresponds to the clinically defined secondary glioblastoma (characterized by a history of prior lower grade diffuse gliomas) and preferentially arises in younger patients; and (iii) glioblastoma NOS, for which full IDH evaluation cannot be performed [13,17]. Primary and secondary GBMs constitute distinct diseases that develop through different genetic pathways and differ in prognosis and therapy response. Primary glioblastomas typically display EGFR overexpression, CDKN2A (p16) deletions, PTEN (MMAC1) mutations, and MDM2 amplification. In contrast, secondary glioblastomas often have TP53 mutations as the earliest alteration [18].

## 4. Barriers of the CNS

The brain was long believed to be widely disregarded by the immune system because of two morphological peculiarities: the absence of lymphatic vessels and the presence of the blood-brain barrier (BBB) [19]. Two physical barriers regulate the moving of immune cells from the blood to the CNS: the BBB formed by the CNS parenchymal microvessels and the blood-cerebrospinal fluid (CSF) barrier formed by the choroid plexuses [20]. The BBB is a selectively permeable barrier composed of endothelial cells, pericytes, and astrocytes, which shields the brain from pathogens, toxins and inflammatory cells within the peripheral circulation [19]. In physiologic conditions, the brain hosts an immunosuppressive milieu that protects the delicate (and non-regenerative) neural tissue from inflammatory insults [4]. Leukocytes entry into the CNS is restricted due to the tight junctions of the BBB at the capillary segment of the vascular arbour. Leukocytes recruitment occurs in postcapillary venules, and three distinct entry routes may be defined: (i) from blood to CSF, across the choroid plexus; (ii) from blood to the subarachnoid space, through meningeal vessels; and (iii) from blood to parenchymal perivascular spaces [21]. In experimental autoimmune encephalomyelitis, it has been demonstrated that mononuclear cells’ diapedesis occurs via the transendothelial process of the cerebral venules, keeping the endothelial tight junctions intact [22]. Also, CSF circulation has been revised by the discoveries over the glymphatic system, involving paravascular routes where fluids are freely exchanged between the brain’s cerebral interstitial fluid (ISF) and CSF, without crossing the endothelial cell layer [23,24,25]. Meningeal lymphatic vessels receive CSF from the adjacent subarachnoid space and ISF via the glymphatic system, then transporting the fluids into deep cervical lymph nodes via foramina at the skull base [26]. Thus, the BBB is not designed to keep leukocytes out of the brain but contributes to regulating immunoregulatory cells and molecules in the CNS microenvironment [27]. Also, the CNS immune privilege may be explained by the lack of dendritic cells (DCs) or other resident antigen-presenting cells (APCs) capable of conveying antigens to lymph nodes outside the CNS parenchyma. This characteristic makes the CNS virtually resilient to constructing immune reactions [28].

## 5. Tumor Microenvironment in Glioblastoma

One of the main explanations for treatment resistance and tumour recurrence in GBM resides in the big changes within the tumour microenvironment (TME) [29]. TME is made up of numerous cell types: (i) tissue-resident cells such as neurons and astrocytes; (ii) myeloid cells such as resident microglia; (iii) bone marrow-derived macrophages (BMDMs), bone marrow-derived DCs and neutrophils; (iv) other immune cells as lymphoid cells; (v) endothelial cells, pericytes, and fibroblasts. All these cells are surrounded by a distinctive extra-cellular matrix (ECM) [30,31,32,33]. Bidirectional interaction between cells and their microenvironment is crucial for both normal tissue homeostasis and tumour growth. In particular, tumour cells interact with the associated stroma and influence disease initiation, progression, and patient outcome [34]. A state of immunosuppression characterizes GBM’s TME, thanks to the secretion of several cytokines by tumour cells, microglia, and tumour-associated macrophages (TAMs) [32]. GBM cells produce cytokines, chemokines, growth factors and ECM modifying enzymes, extracellular vesicles and proteins to construct a favourable tumour microenvironment [29]. Also, cells in TME interact with each other and with the neoplastic cells through different suppressor receptors like PD-1, CTLA-4, CD70 and gangliosides that increase the tumour immune escaping [35,36]. This results in: (i) the inhibition of the immune response; (ii) the activation of FoxP3+ regulatory T-cells (Tregs); (iii) the suppression of NK activity and T-cell activation; (iv) the induction of T-cell apoptosis; (v) the switch to an M2 immunosuppressive phenotype of TAMs; and (vi) the downregulation of MHC expression [37,38].

A crucial point of the brain’s immune privilege is linked to the resident cells’ intrinsic capacity to induce apoptosis of infiltrating T-cells. This process is driven especially by adult astrocytes representing the first line of defence of the BBB against the entry of activated T-cells in CNS [39,40]. Indeed, astrocytes express death-ligand CD95L (APO-1L/FasL) to kill CD95+ infiltrating T-cells [41,42]. In gliomas, the BBB is compromised, enabling the infiltration of immune cells [29,43].

### 5.1. Cells in GBM TME

#### 5.1.1. Tumour-Infiltrating Lymphocytes-TILs

The majority of the Tumor-infiltrating lymphocytes (TILs) are represented by CD8+ CTLs, CD4+ T helper cells, and by regulatory CD4+/FoxP3+ T-cells (Tregs) [44,45].

CD3+ and CD8+ T-cell infiltration in GBM’s TME increases from low-grade to high-grade tumors, maybe due to the disruption of the BBB, and at the same time it has been correlated to better patients’ prognosis, independently from age, MGMT promoter methylation, and post-operative treatment [44,46].

In general, GBM induces a state of global T-cell impairment and functional immunosuppression, exploiting several different mechanisms such as senescence, tolerance, anergy, exhaustion, and ignorance [45]. Indeed, GBM patients display a reduction of systemic CD4+ T-cells levels together with an upregulation of inhibiting receptors, such as CTLA-4, CD73, and CD39, leading to a decrease in T-cells activity [44].

#### 5.1.2. Regulatory T Cells-Tregs

Immune tolerance is mediated mainly by regulatory T-cells (Tregs), immunosuppressive T-cells, which negatively module the immune response and protect against autoimmunity [47,48,49].

Two types of Tregs with different origins are present in CNS: the thymus-derived natural Tregs (nTregs) that account for most of the brain-tumour resident Tregs and the induced Treg (iTregs) [50,51,52]. In particular, GBM tumour cells recruit nTregs [51,53]. Tregs drives tumour immune microenvironment, tumour progression and patient outcome [50,51,52]. Indeed, GBM immune-escape ability is associated with the presence of Tregs, and their number is related to glioma’s grade. However, exploited mechanisms have been only partially understood [48,49,54]. Several studies have demonstrated how a large amount of CD4+ cells is represented by Tregs, which downregulate the IFNγ-based antitumour response thanks to the production of Th-2 polarizing cytokines, such as IL-10 and TGFβ [47,55,56,57,58]. TGF-βs (β1 and β2) may hamper T-cell recruitment in CNS through the downregulation of ICAM-1 and VCAM-1; this finding opens the possibility to use anti-TGF-βs drugs to enhance immunotherapy efficacy [46].

Tregs recruitment occurs through the binding of CCR4 on Tregs surface and CCL22 secreted by GBM tumour cells and CD163+ TAMs, under the induction of tumour-derived CCL20 and osteoprotegerin [59,60]. Another mechanism involved in Tregs chemotaxis is the expression of the tumour-enzyme indoleamine 2,3 dioxygenase (IDO), which has been associated with tumour progression and lower survival-rates [61,62].

Several target therapies against Tregs have been tested in combination with standard chemotherapy, such as mAb against IL-2Rα (CD25) and CTLA-4, cytokines inhibitors or GITR agonists, up-regulated in Treg and activated T-cells. However, only low levels of response have been reported with the administration of these innovative drugs [47,57,58,63,64,65,66,67].

#### 5.1.3. CAR-T

Recently, the broad term “T cell exhaustion” has been used to describe not only the response of T cells to chronic antigen stimulation in the setting of chronic viral infection but also in response to tumours. T-cell exhaustion is tightly linked to the TME and the production of several concomitant key-factors: (i) the upregulation of inhibiting receptors (PD-1, Tim-3, CD39, TIGIT, Lag-3 and CTLA-4); (ii) the presence of cytokines’ dysfunction, with high levels of immunosuppressive soluble mediators (IL-10, TGF-B, PGE2 and CSF-1); and (iii) the recruitment of immunosuppressive cell species, such as MDSC or Tregs [68,69].

A therapy based on genetically engineered T-cells with Chimeric Antigen Receptor (CAR) has been developed to increase the tumour immunogenicity. CARs are artificial fusion proteins engineered to bind to specific tumour antigens via a single-chain fragment variable (scFv) [70,71] This allows inducing an anti-tumour T-cell response, overcoming the low tumour mutational load, the defective neoantigens presentation, and the lack of immune priming in GBM [72,73,74].

Significant results were obtained by anti-CD19 CAR-T cells in the context of hematologic tumours [75,76,77]. Pre-clinical and early phase clinical trials have been conducted on several brain tumour targets, such as IL13Rα2, EGFRvIII, HER2, EphA2, GD2, B7-H3, chlorotoxin, and CD70. In particular, three early phase trials targeting IL13Rα2, Her2/CMV, and EGFRvIII have been performed, reporting good safety and tolerability rates, with a promising anti-tumour response [78,79,80,81]. Furthermore, HER2, EGFRvIII, and IL-13 receptor α 2 (IL-13 Rα2) are also exposed on the surface of cancer stem cells (CSCs), paving the way for new possible application of CAR-T therapy in cancer immunology [82].

A more profound comprehension of cellular heterogeneity and the possibility of combining other immunotherapeutic approaches may overcome CAR-T tumour resistance.

#### 5.1.4. Microglia and Tumour-Associated Macrophages-TAMs

During embryonic life, some progenitor cells from the yolk sac migrate into the CNS and differentiate into parenchymal microglia, maintained by self-renewal during adult life [83,84,85,86].

Microglia preserves neuroimmune homeostasis, supporting the axon’s development and contributing to the elimination of apoptotic cells, but also plays a pivot role in cancer setting [87].

In GBM, tumour-associated macrophages (TAMs) may have two different origins: (i) tumour-infiltrating monocytes, which derive from the hematopoietic stem cells (bone marrow-derived macrophages-BMDMs); and (ii) tissue-resident macrophages. Among this latter group, two further subpopulations can be recognized: (i) the above mentioned parenchymal microglia; and (ii) the peripheral brain macrophages located in the meninges, choroid plexuses and the perivascular sites [35,83,84,85,86,87,88,89].

According to the TAMs’ origin, GBM’s TME reveals different expression levels of proteins involved in antigen presentation and cytokines production, such as IL-1 [90,91].

This could explain the ability of GBM’s TME to select functionally different TAMs populations that may play a different role in the context of the tumour growth, as demonstrated in other malignancies, such as lung and breast cancer [92,93].

In GBM tissue, TAMs account for about one-third of TME’s cells. They have been shown to represent a potentially powerful target for targeted therapy [84,94]. Indeed, the number of TAMs is related directly to tumour grade, and inversely to patients’ survival [14,34,95].

To understand the functional role of the different TAMs populations, one of the most significant challenges to face is represented by the segregation between microglia and BMDMs, through specific biomarkers [96,97,98].

These two different cell populations share positivity for CD68, CXCR1, F4/80, and IBA1 [99]. Though, transcriptional profiling and in vivo 2-photon microscopy clearly demonstrated the profound molecular differences between these two populations [83,84,100]. In the CNS, microglia commonly does not express CD45; however, it expresses CD45 in inflammatory conditions, becoming hardly distinguishable from BMDMs [35,101]. Likewise, CXCR3 is expressed only by microglia; however, it is also expressed by BMDMs within brain tumours [83].

Conversely, microglia could be discriminated by the positivity for TMEM19, P2RY12, Sall1, and by the low expression of CD206 [102,103,104]. Instead, BMDMs expose on their surface CD44, CD169, and high expression of CD45 and CD206 [84,105].

The binding of microglia to immunogenic antigens like lipopolysaccharide through specific surface receptors, such as TLRs, NODs, SR, leads to its activation towards a pro-inflammatory M1 phenotype, characterized by: (i) the activation of enzymes, such as NADPH oxidase and inducible nitric oxide synthase (iNOS); (ii) the increased transcription of STAT1, NF-Kβ, TNF-α; (iii) the secretion of pro-inflammatory chemokines and cytokines; (iv) the acquisition of antigen presentation capacity, thanks to the expression of MHCI and costimulatory molecules like CD40, CD80, and CD86 [30,106,107,108].

The transition to an M2-like anti-inflammatory phenotype is mediated by IL4 and IL13 produced by Th2 lymphocytes, leading to the activation of STAT6, TGF-β and IL10, together with CCL15, -17, -22, and -24 [30].

M2 polarized macrophages are further sub-classified into M2a, M2b, and M2c phenotypes, and express both general (CD68, CD11b, and IBA1) and M2-related markers (CD163, CD204 and CD206) [109,110].

There is a bidirectional interaction between tumour cells and M2c cells. GBM cells produce IL10, TGF-β and glucocorticoids, stimulating the expansion of the M2c population, which in turn promotes the proliferation of tumour cells. Hence, GBM’s TAMs acquire mainly the M2 polarization, and an unbalance interchange of M1-M2 can be detected [109,111,112,113].

These assumptions make immunosuppressive pro-tumoral M2 macrophages a potential therapeutic target, as demonstrated by oncolytic virotherapy, able to revert M2 to M1 phenotype [99].

This innovative approach is based on the injection of engineered viruses into the systemic circulation or directly at the tumour site, inducing the lysis of the tumoral cells [114]. This results in spreading tumoral neoantigens and damage factors that produce a concomitant innate and adaptive immune activation directed against the tumour [114,115,116].

Indeed, some pioneering clinical trials showed how oncolytic viruses could revert to “warm” the immunologically “cold” GBM’s microenvironment, and increase the response of the tumour to checkpoint inhibitors, as well as to the standard treatments [66,117,118].

#### 5.1.5. Dendritic Cells—DCs

Dendritic cells (DCs) are ubiquitous specialized antigen-presenting cells (APCs) that coordinate several immune responses, from self-tolerance to fighting infections [119].

In a cancer setting, mature DCs can show two different behaviours: immunostimulatory or immunosuppressive. Indeed, DCs can drive the immune response against tumour cells. In particular, DCs promote both the cell-mediated anti-tumour T-cells activation and humoral response [120,121,122]. In turn, tumour cells try to shield themselves, producing immunosuppressive molecules able to change the TME composition, hindering DCs activation and migration, and finally evading immune control [121,123]. For example, cancer cells secrete Fibrinogen-like Protein 2 (FGL2) that antagonizes GM-CSF and blocks the cDC1 activation, escaping from immune-control [124,125].

Ovarian cancer represents a peculiar model to study DCs behaviour; in this tumour, DCs show an immunostimulatory phenotype in the early stages, that becomes immunosuppressive in the later stages. In particular, cancer progression is promoted by the lack of an anti-tumour T-cells response lead by DCs [6,126]. On the other hand, tumour-associated DCs (TADCs) can help tumour expansion, promoting new vessel formation and genomic instability [127,128].

According to their different immunophenotypic profile, distinct DCs populations have been found in the TME of different solid tumours, such as conventional DC1 (cDC1) and DC2 (cDC2), plasmacytoid DCs (pDCs), and monocyte-derived DCs (Mo-DCs) [127,129,130].

Each DCs subgroup develops under the control of a specific repertoire of transcription factors, such as IRF8, IRF4, and E2-2. In particular, cDC1 are IRF8-dependent, express CD141 and CCR7, and migrate from the tumour site to draining lymph nodes stimulating the activation of CD8+ T anti-tumoral cells. The presence of these cells in the TME is related to immune-mediated rejection and may represent a specific target to exploit for DC-based tumour therapies [127,131,132]. Indeed, in melanoma patients, cDC1 are recruited by distinct chemokines like Flt3L, CCL5, and XCL1 produced by NK-cells, and are associated with favourable response to anti-PD1 immunotherapy and better outcome [133,134]. Instead, cDC2 are IRF4-dependent, express CD11b, and lead the activation of Th17 cells, as in the vaccination of mice bearing Lewis lung carcinoma, where significant tumour growth decrease has been registered [127,131,135]. A further subtype of DCs is represented by pDCs, which are E2-2 dependent, express CD123 and are activated by viral infections, producing high quantities of interferon and cytokines. Finally, Mo-DCs are CCR2-dependent and originate from bone marrow-derived Ly6Chi monocytes; Mo-DCs perform antigen processing and presentation functions, even with an inefficient activation of T-cells [136,137].

The organization of the DCs in CNS has not been fully elucidated, and many issues on their function in the context of GBM’s TME remain still unsolved.

Flores et al. demonstrated that HSC CCR2+ migrate from bone-marrow to intracranial PD-L1 resistant brain tumours (GBM and medulloblastoma), giving rise to APCs like Mo-DCs, which stimulate the CD8+ T-response against tumour cells. These findings may pave the way for the DC-therapy as an alternative strategy to treat checkpoint blockade resistant tumours [138].

In support of this, some mouse models studies showed that the use of TLR3 and Flt3L enhances DCs’ activation and, in combination with checkpoint blockade therapy, anti-tumour response is improved [139,140].

Wang et al. have demonstrated how DCs exposed to GBM antigens improve the expression of Nrf2, known to correlate to an immunosuppressive state (with an increase in IL-10 and a reduction in CD80, CD86, and IL12). Hence, the inhibition of Nrf2 could intensify the activity of DCs and T-cells against tumour cells [141].

Based on the above findings, the goal of DCs-based therapies would be to promote cDC1 in amplifying tumour-recognition and eradicating tumour cells by the immune system.

This therapeutic strategy is based on loading DCs with GBM-key antigens, thus activating a specific immune response against them [142]. Peptides or tumour lysates together with immune-modulators are used to create vaccines that promote a durable immune activation against GBM [67,142]. Several pre-clinical and clinical studies demonstrated an increase in immunological memory and overall survival, with a good tolerability profile [143,144,145]. However, further clinical trials are needed to evaluate better the efficacy of DC-based therapies in GBM’s treatment.

### 5.2. Molecules in GBM’s TME

In GBM’s microenvironment, different mediators are secreted by several cell subtypes, which constitute a complex network between immune and neoplastic compartments, such as: (i) cytokines (IL-6, IL-10, VEGF, TGFβ, M-CSF, GM-CSF, bFGF); (ii) prostaglandins (PGE2); (iii) chemokines (CCL20 and CXCL8); (iv) reactive oxygen species; and (v) cellular components like gangliosides [146,147,148].

TME is supported and modelled by tumour cells through growth factors, ECM modifying enzymes, and extracellular vesicles carrying RNA and proteins [29].

In particular, GBM cells secrete many factors like hepatocyte growth factor (HGF), monocyte chemotactic proteins (MCPs), glial neurotrophic derived factors (GNDF), macrophage colony-stimulating factor (M-CSF), granulocyte-macrophage colony-stimulating factor (GM-CSF), and the transforming growth factor-β (TGF-β) [14,149].

Furthermore, GBM cells recruit and polarize BMDMs in an M2 immunosuppressive phenotype thanks to the secretion of soluble factors (such as osteopontin, postin, and kynurenine), and contribute to the impairment of T-cell function and the establishment of an immunosuppressive state [150,151,152,153].

In turn, tumour-associated macrophages (TAMs) and microglia represent a rich source of chemokines, ECM constituents, angiogenic molecules and growth factors capable of increasing and supporting GBM cells proliferation and infiltration [30].

Novel therapeutic opportunities may derive from discovering the essential pathways and molecules involved in the crosstalk between GBM cells and their microenvironment.

#### 5.2.1. Transforming Growth Factor-β-TGF-β

GBM cells produce the glioblastoma-derived T-cell suppressor factor (G-TsF), a member of the TGF-β family re-named as TGF-β2, which induces immunosuppression by: (i) blocking T-cells activation and proliferation; (ii) downregulating MHC class II expression on CD4+ T-cells; (iii) inhibiting IL-2 production; (iv) suppressing NK-cells activity; and (v) promoting the activation of FoxP3+ regulatory T-cells (Tregs) [33,37,38]. Accordingly, in murine models infected with lymphocytic choriomeningitis virus (LCMV), the injection of recombinant G-TsF/TGF-β2 inhibits the generation of virus-specific cytotoxic T-cells [33,38,154].

TGF-β promotes tumour growth and invasion by supporting and stimulating GBM stem cells’ migration and enhancing angiogenesis; in particular, it inhibits tumorigenesis in early stages, and promotes tumour growth in later stages [33,38,154].

All these mechanisms contribute to the impairment of tumour immune surveillance and open the possibility of immunotherapeutic strategies based on TGF-β signalling pathway inhibition. [155,156].

#### 5.2.2. Prostaglandin E2-PGE2

Prostaglandin E2 (PGE2) is a prostaglandin produced by COX2 and plays an essential role in the angiogenesis of glial tumours, particularly in GBM, which is a highly vascularized tumour with angiogenesis-dependent growth [157]. Indeed, the increased expression of COX-2 has been correlated with gliomas’ grade [158].

In GBM’s microenvironment, PGE2 concentration is not high enough to suppress T-cell functions on its own. However, PGE2 works together with TGF-β, transforming DCs into regulatory cells and suppressing T-cell proliferation [38].

New antiangiogenetic therapies are based on the oral administration of specific Cox-2 inhibitors that reduce angiogenesis and tumour growth combined with low-dose scheduling of chemotherapeutic drugs [159].

#### 5.2.3. Interleukin-10-IL-10

IL-10 is secreted by immune cells as well as by GBM cells and enhances tumour growth, inhibits the production of interferon-γ (IFN-γ) and tumour necrosis factor-α (TNF-α) by the immune system, and suppresses the antigen presentation process [33,160,161].

In vitro, it has been demonstrated that IL-10 upregulates *KPNA2* (a gene coding for nuclear import of proteins); at the same time, knockdown experiments showed that glioma cell growth and invasion were significantly reduced, suggesting IL-10 as a potential target for glioma patients’ treatment [162].

#### 5.2.4. Colony Stimulating Factor 1-CSF-1

Colony-stimulating factor 1 (CSF-1) is a crucial chemokine for TAMs differentiation and survival. CSF-1 works in combination with EGFR, promoting GBM cells invasion [163].

The inhibition of CSF-1 and EGFR prevents and reduces tumour invasion, resulting in a significant improvement in patients’ survival and clinical outcome [94,164].

#### 5.2.5. Cluster of Differentiation 38-CD38

CD38 is an ectoenzyme involved in TAMs promotion and initiation, which is present on the surface of one-third of the cells [165,166].

The selective inhibition of CD38 with a monoclonal FDA-approved antibody called daratumumab (DARA) enhances tumour immune recognition and reduces tumour growth in vitro and in GBM mouse models. In particular, the association of TMZ and DARA produces better results in terms of anti-tumoral apoptotic effects than TMZ alone [167]. In light of these data, future experiments will be necessary to understand better the role of CD38 in GBM’s microenvironment [168].

#### 5.2.6. Programmed Death Ligand 1-PD-L1

In physiological conditions, the programmed death-1 receptor (PD-1)/Programmed death ligand 1 (PD-L1) axis plays an active role in immune homeostasis and prevents autoimmune response thanks to the activation of Treg cells and the inhibition of aberrant self-reactive T-cells [169,170].

PD-L1 belongs, together with PD-L2, to the B7 proteins family. Both bind to PD-1, a receptor of the CD28/cytotoxic T-lymphocyte-associated protein 4 (CTLA-4) family [171]. The role of this axis as therapeutic target is well known in several solid malignancies [172,173,174].

GBM cells can upregulate the activity of the co-inhibitory pathway B7-CD28 to induce TME immunosuppression and escape from immune control [175].

In fact, PD-L1 is expressed on the surface of GBM cells, tumour infiltrating myeloid cells (TIMs), B-cells, and CNS cells. Recent data suggest that TIMs show the largest amount of PD-L1 molecules, promoted by IL-10 secreted by tumour cells [176,177,178].

The binding of PD-L1 with PD-1 leads to the activation of an immunosuppressive pathway in which the tyrosine phosphatase SHP2 dephosphorylates Zap70, downregulating the cytotoxic activity of lymphocytes and, simultaneously, enhancing the migration ability of GBM cells [176].

The activity of PD-L1 is undoubtedly complex, as shown by the numerous receptors to which it binds, such as PD-1, CD28, CD80 and CTLA-4. However, this mechanism grants to target multiple immune-pathway simultaneously, thanks to the inhibition of only PD-L1 [171].

Instead, PD-L2 is expressed primarily by DCs rather than tumour cells, is stimulated by several TME cytokines, such as IL-15 and IL-7, and binds only to PD-1. This protein is less characterized in GBM than PD-L1, but it has been revealing as a prognostic biomarker. In particular, the overexpression of PD-L2 is related to worse overall survival in GBM patients [171,179].

Conversely, relevant studies also showed how PD-L1 expression in GBM’s microenvironment could have a prognostic impact; in particular, if the expression is high in neurons and low in GBM cells, patients show better outcomes [180]. In contrast, increased expression in glioma cells is related to high tumour grade and worse patient outcomes [171,181].

According to these data, a new therapy based on the inhibition of the signalling cascade mediated by PD-1/PD-L1 has been developed to enhance GBM antigens’ recognition by the self-immune system [182]. Preclinical studies showed a significant regression of tumour mass and a longer survival time in GBM mouse models [176]. In patients with recurrent GBM, monoclonal antibodies against PD-1 and PD-L1 are now being tested within clinical trials. Therapies based on PD-1 inhibitors, such as pembrolizumab and nivolumab, have shown promising results in non-small lung cancer and melanoma but still represent a poor option in GBM due to the peculiar, high immunosuppressive state of this tumour [183,184,185,186]. Similarly, PD-L1 inhibitors, such as durvolumab or atezolizumab, have reported low efficacy in patients with GBM, with only minimal survival improvement for a subgroup of GBM patients with mismatch repair deficiency [187,188]. However, preclinical studies showed that PD-L1 pathway remains immunologically significant, paving the way for new therapeutic strategies [177,178].

#### 5.2.7. Cytotoxic T-Lymphocyte-Associated Protein 4-CTLA-4

CTLA-4 is an immunosuppressive receptor located on the surface of T-cells, which blocks the initiation of the adaptative immune response, binding to specific costimulatory molecules (CD80 and CD86) expressed by APCs and essential for the naïve and memory T-cell activation. Also, the CTLA-4 is involved in the inhibition of T-cells by Tregs to preserve peripheral tolerance [189].

Hence, the inhibition of CTLA-4 unlocks CD4+ and CD8+ effector cells and, at the same time, decreases Tregs activity, supporting and promoting the initiation of an antitumor T-cell response [190,191].

In gliomas, CTLA-4 overexpression is associated with higher tumour grade and worse clinical outcome [192].

To date, ipilimumab, an anti-CTLA-4 monoclonal antibody, has been administered to GBM patients, reporting tolerability troubles [193].

#### 5.2.8. T-Cell Immunoglobulin Mucin-3-TIM-3

T-cell immunoglobulin mucin-3 (TIM3) is a molecule located on the surface of various immune cells, such as Th1 CD4+ and CD8+ cytotoxic T-cells, DCs, NK-cells, and monocytes. The binding of TIM3 with galectin-9, HMGB1 and CEACAM1 leads to immunosuppression and the dysregulation of the innate and adaptative responses.

In cancer, TIM3 may represent an important immunotherapeutic target, particularly in the context of GBM resistance to previous checkpoint inhibition therapies [194,195].

#### 5.2.9. Lymphocyte-Activation Gene 3-LAG-3

Lymphocyte-activation gene 3 (LAG-3) is a molecule presented on the surface of CD4+ and CD8+ T-cells, NK-cells and B-cells [196,197].

LAG3 prevents immune cells’ initiation, binding to crucial receptors, such as MHC-II or LSECtin, to activate CD4+ T-cells or CD8+ T-cell/NK-cells respectively [198].

Studies in humans and mouse models demonstrate that LAG-3 inhibition contrasts GBM progression with particular efficacy in the early stages of treatment [199]. Furthermore, the concomitant synergic blockade of LAG-3 and PD-1 has revealed a more potent anti-tumoral effect than the anti-PD-1 therapy alone in several cancer models [200,201].

These data support the possibility of administering anti-LAG-3 therapy in combination with other checkpoint blockade strategies to achieve a better response in GBM patients.

#### 5.2.10. Cluster of Differentiation 70-CD70

CD70 is a molecule presented on the surface of B- and T-cells, that activates other T- and B- cells or NK-cells, interacting with the glycoprotein CD27 [202].

However, GBM cells may express CD70, promoting immunosuppression through tumour-induced T-cells apoptosis and Tregs’ activation [202,203].

Interestingly, Varlilumab, an anti-CD27 agonist, is now administered in ongoing clinical trials in combination with nivolumab [204].

#### 5.2.11. Gangliosides

Gangliosides are a large family of glycolipids expressed in abundance by GBM cells and represent potential targets for GBM immunotherapy [205].

O-acetylated-GD2 represents the first effective ganglioside target discovered. Indeed, monoclonal antibodies against O-acetylated-GD2, such as Dinutiximab, demonstrated to reduce tumour cells proliferation, both in vivo and in vitro models [206,207]. Moreover, a truncated form of O-acetylated-GD2, delivered by genetically engineered mesenchymal stromal/stem cells, mediates immunoselective recognition of GD2-positive tumours, obtaining a potent anti-tumour activity [208].

## 6. Cancer Stem Cells and Tumor Microenvironment

Cancer stem cells (CSCs) are involved in tumour initiation, relapse, metastasis and poor survival in multiple malignancies, including GBM [209]. Gliomas contain self-renewing, tumorigenic cells that have been shown to express undifferentiated neural cells markers, such as nestin [31,210,211].

Clonogenic cells with an ambiguous “stem-like” phenotype were found in cortical gliomas bearing p53 mutations. These cells express both neuronal and glial cytoskeletal markers and reveal an epigenetic modulation of anti-apoptotic and de-differentiation genes [31,212,213].

Evidence that tumorigenic stem-like precursor cells resemble glial progenitors, both in markers expression and migratory properties, suggest that glial progenitors that populate the adult CNS represent the source of gliomas [214,215,216].

These cells are capable of self-renewal, proliferation, and differentiation into tumour cells with multiple expression markers, resulting in the building of a heterogeneous tumour [217].

### 6.1. Glioblastoma’s Molecular Subtypes

Different GBM subtypes are associated with different “cells of origin”, distinguished by peculiar alterations in specific genes, such as EGFR, NF1, and PDGFRA/IDH1 [90,218,219]. Dysregulation of these genes and specific transcription factors (TFs), such as POU3F2, SOX2, SALL2, and OLIG2, drives stem-like cells differentiation into three particular GBM subtypes: the proneural, classical, and mesenchymal [220]. Moreover, different TFs, such as Musashi1 and BIM1, are associated with the expression of the podocalyxin-like protein (PODXL) and the presence of undifferentiated GBM stem-like cells, thus correlating with high glioma grade and worse patients’ outcome [221].

As a result of this heterogeneity, GBM may present a combination of different molecular subtypes within the same tumour in variable percentages, reflecting different responses to therapy: in particular, the classical subtype has the best outcome, whereas the proneural one has the worst [219].

### 6.2. Glioblastoma Stem Cells’ Niches

GBM CSCs are thought to reside inside microanatomical structures within the tumour (the so-called “tumour niches”), where are maintained and protected from the immune system [222]. Three niches are described within a tumour: the perivascular, the necrotic/hypoxic and the invasive niche. These are functionally distinct, interchangeable, and contain infiltrating myeloid cells, suggesting a close connection and crosstalk between glioma stem cells and the microenvironment [223,224].

These cells play a crucial role in recurrence and disease progression, as demonstrated by their resistance to chemo- and radiotherapy [225,226,227]. Hence, it was recently proposed to target the stem cell-like component of GBM to overcome this issue [228,229,230,231].

GBM CSCs are regulated by intrinsic factors, such as genetic, epigenetic, and metabolic modifications, and by extrinsic factors, including interactions with the TME and the immune system [31].

Transcriptional and epigenetic control, together with several genetic alterations, determine the complex clonal diversity that characterizes GMB [231]. Intertumoral heterogeneity in GBM results from distinct differentiation lineages of different precursor cells, suggesting the presence of multiple stem cell-like populations [219,231].

Similarly, metabolism affects heterogeneity in GBM; indeed, the hypoxic tumour microenvironment supports CSCs self-renewal, proliferation, and survival. IDH1 mutations represent an interesting link between genetics and metabolism in GBM, causing accumulation of 2-hydroxyglutarate, thus promoting aberrant hypermethylation of DNA and histones, and dedifferentiation [232,233,234,235,236,237,238].

CSCs niches result from the complex microenvironmental, metabolic, genetic, and epigenetic alterations that promote tumour growth by activating specific pathways, such as Nf-kB, Notch, BMP and Wnt signalling [234,239,240,241,242,243,244].

CSCs of perivascular niches exploit Notch signalling to interact with the endothelial cells and promote neoangiogenesis through VEGF, supporting infiltrative growth. Moreover, GBM CSCs also play a direct role in building new vessels by transdifferentiation into pericytes [226,245,246,247,248,249].

CSCs stimulate the activation of FoxP3+ regulatory T-cells, which produce IL-10 and TGF-β, that in turn differentiate TAMs into an immunosuppressive M2 phenotype [250,251,252]. This immunosuppressive effect is amplified by the ability of the CSCs to promote the recruitment and attraction of microglia, even more than the differentiated neoplastic cells [222].

Also, CSCs inhibit T-cells proliferation and effector responses, and induce T-cells apoptosis through PD-1, PD-L1, and soluble Galectin-3 [31,250].

These data open future possibilities to combine anti-CSCs drugs with other immunotherapeutic strategies.

## 7. Future Perspectives

To date, several clinical trials are still ongoing, and different therapeutic approaches against TME have been tested, with alternating achievements. Indeed, improved outcomes were observed only in an extremely selected and very restricted patient population.

**Figure 1:**
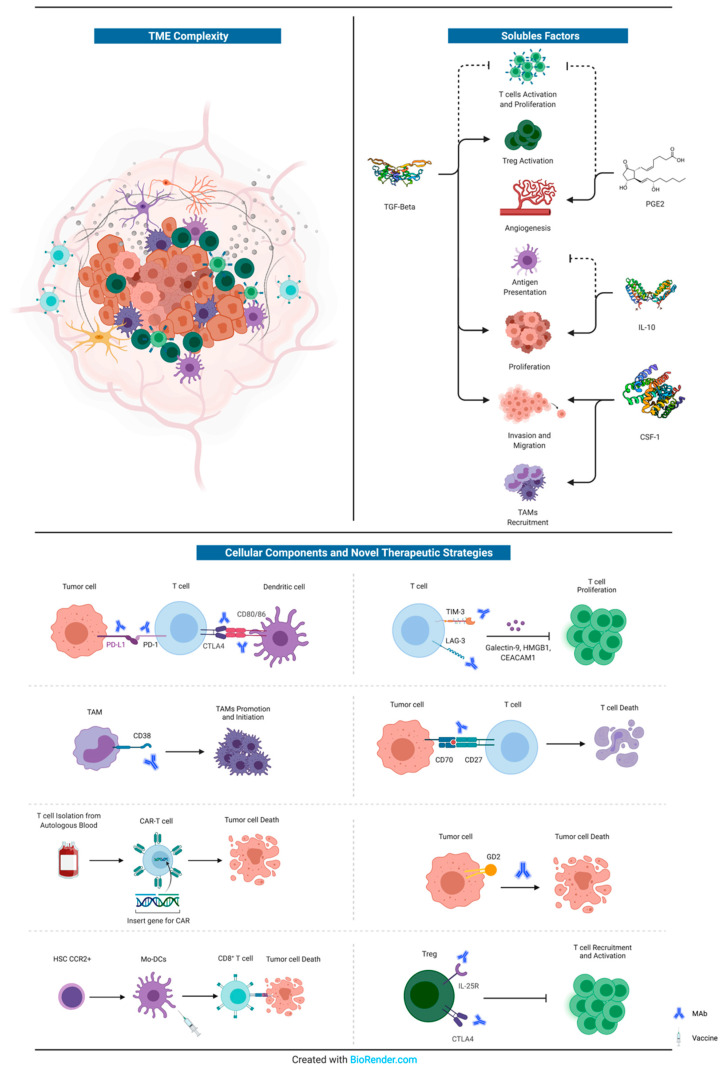
Schematic representation of the complexity of GBM’s microenvironment. The principal soluble factors with crucial roles in GBM proliferation, migration, neoangiogenesis, and immune-escape are represented in the upper right corner. Instead, a summary of the cellular components of GBM’s immune microenvironment that interact with tumour cells is reported at the bottom, together with novel therapeutic strategies (monoclonal antibodies or vaccines). For further information, see text. MAb: monoclonal antibody.

The most promising and innovative approaches (other than those directed against GBM tumour cells) are represented by: (i) Varlilumab, an anti-CD27 agonist, administered in combination with Nivolumab [204]; (ii) Daratumumab, an anti-38 agent, already approved for the treatment of multiple myeloma, but still in pre-clinical phase for GBM [168]; (iii) CAR-T cell therapy, even if we are only at the dawn of the application of this innovative technique for GBM treatment [71]; (iv) Dinutiximab, an anti-GD2 agent, previously tested against neuroblastoma [207]; anti-LAG3 (BMS-986016) or anti CD137 (BMS-663513) plus anti-PD1, but still in phase I trial [199]; (v) immune checkpoint inhibitors, such as Nivolumab [253], Pembrolizumab [193,254], Durvalumab [255], Ipilimumab [193] and Atezolizumab [256], alone or in combination with antiangiogenics drugs (i.e., bevacizumab) [257] or dendritic cells vaccines [67,142,143,144,258,259,260]. All these novel therapeutic strategies are summarized in Figure 1.

## 8. Conclusions

Glioblastoma remains a complex and deadly disease. Deciphering of the multifaced bidirectional network between tumour cells and tumour microenvironment clearly represents the future of translational research, as demonstrated by the several molecular interactions under investigation. To date, some novel targeted approaches are under study; however, a long and winding road is still to be travelled before obtaining a game-changing therapy. Different skills with different viewpoints need to be merged to speed up this process, and a large amount of complex data must be shared within the entire scientific community. 

## Data Availability

Data sharing not applicable.
